# 

**Published:** 1887-06

**Authors:** 


### OUR ADVERTISERS.

See advertisements of medical colleges in this issue and write for catalogues.

BOOK OF THE DOG.—We have received from the Associated Fanciers, 237 South Eighth street, Philadelphia, a copy of their Dog Buyers' Guide. It contains a finely-executed colored frontispiece, well-drawn engravings of nearly every breed of dog, and all kinds of dog furnishing goods. We should judge that the book cost to produce a great deal more than the price asked—15 cents—and would advise all our readers who are interested in dogs to send for the book.

MELLIN'S FOOD.—Time was when this food or that food, advertised as supplying the want of nature's full supply, had considerable popularity and temporary success. But that all these various preparations contained a large quantity of bulky, indigestible starch-matter was undeniable. Liebig says: "It is no mistake, but a fact, that the usual farinaceous foods are the causes of most of the diseases and of half of the cases of death among babies." Mellin's food, made upon the principles advanced by Liebig, contains no farinaceous, unassimilable matter whatever, and as a nourishing diet for weak stomachs, as a substitute for insufficient nutrition, and as a milk-producer or nursing mothers, it stands unrivaled among prepared foods.

THE FEEDING OF INFANTS DEPRIVED OF THE BREAST MILK.—No physician should recommend a food, as he would not a medicine, without knowing its composition, and the composition of most of the recent dietetic preparations, ending with Carnrick's, has been announced. Carnrick's food contains a large percentage of the solid constituents of milk, the casein of which has been partially digested so as to resemble the casein of human milk in its behavior under the digestive ferment. The other ingredient is stated to be wheat flour subjected to prolonged baking, so that its starch is to a considerable extent converted into dextrine. This food has the advantage of easy preparation in the nursery and easy digestion. Used alone it is sufficiently nutritious for the infant. It will probably supersede some of the older foods of the shops. Poor families, who cannot afford to use it as the sole food, will, according to my observation, find it useful made into a thin gruel and employed in diluting the cow's milk with which these infants are fed.—*By J. Lewis Smith, Clinical Professor of Diseases of Children, Bellevue Hospital Medical College.*

